# Global MicroRNA Expression Profiling Identifies MiR-210 Associated with Tumor Proliferation, Invasion and Poor Clinical Outcome in Breast Cancer

**DOI:** 10.1371/journal.pone.0020980

**Published:** 2011-06-29

**Authors:** Françoise Rothé, Michail Ignatiadis, Carole Chaboteaux, Benjamin Haibe-Kains, Naïma Kheddoumi, Samira Majjaj, Bassam Badran, Hussein Fayyad-Kazan, Christine Desmedt, Adrian L. Harris, Martine Piccart, Christos Sotiriou

**Affiliations:** 1 Translational Research Unit, Jules Bordet Institute, Université Libre de Bruxelles, Brussels, Belgium; 2 Computational Biology and Functional Genomics, Harvard School of Public Health, Dana-Farber Cancer Institute, Boston, Massachusetts, United States of America; 3 Laboratory of Experimental Hematology, Institut Jules Bordet, Université Libre de Bruxelles, Brussels, Belgium; 4 Cancer Research UK Molecular Oncology Laboratories, Weatherall Institute of Molecular Medicine, University of Oxford, John Radcliffe Hospital, Oxford, United Kingdom; Institut de Génomique Fonctionnelle de Lyon, France

## Abstract

**Purpose:**

Aberrant microRNA (miRNA) expression is associated with cancer and has potential diagnostic and prognostic value in various malignancies. In this study, we investigated miRNA profiling as a complementary tool to improve our understanding of breast cancer (BC) biology and to assess whether miRNA expression could predict clinical outcome of BC patients.

**Experimental Design:**

Global miRNA expression profiling using microarray technology was conducted in 56 systemically untreated BC patients who had corresponding mRNA expression profiles available. Results were further confirmed using qRT-PCR in an independent dataset of 89 ER-positive BC patients homogeneously treated with tamoxifen only. MiR-210 functional analyses were performed in MCF7 and MDA-MB-231 BC cell lines using lentiviral transduction.

**Results:**

Estrogen receptor (ER) status, tumor grade and our previously developed gene expression grade index (GGI) were associated with distinct miRNA profiles. Several miRNAs were found to be clinically relevant, including miR-210, its expression being associated with tumor proliferation and differentiation. Furthermore, miR-210 was associated with poor clinical outcome in ER-positive, tamoxifen-treated BC patients. Interestingly, the prognostic performance of miR-210 was similar to several reported multi-gene signatures, highlighting its important role in BC differentiation and tumor progression. Functional analyses in BC cell lines revealed that miR-210 is involved in cell proliferation, migration and invasion.

**Conclusions:**

This integrated analysis combining miRNA and mRNA expression demonstrates that miRNA expression provides additional biological information beyond mRNA expression. Expression of miR-210 is linked to tumor proliferation and appears to be a strong potential biomarker of clinical outcome in BC.

## Introduction

MicroRNAs (miRNAs) are evolutionary conserved, small non-coding RNA molecules that negatively regulate gene expression. The single-stranded mature miRNA is 19–25 nucleotides long and derives from the cleavage of a longer precursor containing a hairpin structure. Post-transcriptional gene silencing by miRNAs occurs through the translational inhibition of the targeted mRNAs or their specific cleavage [1]. Computational analyses indicate that a unique miRNA can regulate hundreds of genes, underscoring the potential influence of miRNAs on almost every cellular pathway. MiRNAs have been shown to regulate various biological processes such as development, differentiation and proliferation [2].

Recent studies have demonstrated that mutations in miRNAs or their aberrant expression are associated with diverse human diseases, including cancers, suggesting that miRNAs may act as oncogenes or tumor suppressor genes. MiRNA genes are frequently located at fragile sites and cancer-associated genomic regions. Recently, miRNA expression signatures have emerged from several studies. Iorio et al. identified a signature that could discriminate normal and breast tumor tissues [3]. Recent findings have also linked deregulated miRNA expression to tumor metastasis in breast cancer (BC) cells [4],[5]. These results suggest that aberrant miRNA expression may be important for the pathogenesis of this malignancy. In addition to being potential diagnostic markers, the role of miRNAs in cancer prognosis has also been highlighted. Indeed, several miRNAs were reported to be associated with the clinical outcome of patients with chronic lymphocytic leukaemia [6], lung [7], [8] and ovarian cancers [9].

Technologies such as microarray have improved our understanding of BC biology, but also disease classification and prognostication [10]–[12]. Indeed, gene expression profiling studies have demonstrated that breast tumors can be divided into at least four clinically relevant molecular subtypes, each with distinct disease outcomes: the predominantly estrogen-receptor (ER)-negative, progesterone-negative, HER-2-negative, basal-like subtype, the HER2/neu-positive subtype and at least the two ER-positive, luminal-like A and B subtypes characterized by their differences in proliferation rate [11]–[12]. These results suggest a biological basis for the clinical heterogeneity of BC.

Our group recently identified a gene expression grade index (GGI), which mainly reflects tumor proliferation and differentiation [13]. This 97-gene index reclassifies patients with histologic grade 2 tumors, a clinically problematic tumor type, into two subgroups with distinct clinical outcomes similar to histological grade 1 and 3 tumors respectively, improving the accuracy of tumor grading and therefore its prognostic value [13]. The GGI can also identify two clinically distinct ER-positive molecular subtypes, demonstrating the importance of proliferation-related genes in predicting prognosis in ER-positive BC patients [14]. Finally, proliferation appears to be one of the most prominent prognostic factor in BC, recapitulating the prognostic power of several first generation gene prognostic signatures, and highlighting the clinical importance of proliferation-related genes for BC prognosis [15].

Considering the importance of miRNAs in carcinogenesis, we sought to investigate miRNA profiling as a complementary tool to improve our understanding of BC biology and its prognostication. To this end, we analyzed miRNA and gene expression profiles from the same BC cohort in order to integrate the information gained from miRNA profiling with that obtained from the microarray gene expression profiling of protein-coding genes. We also thought to identify miRNAs associated with established clinical/pathologic variables as well as with the previously reported GGI. Finally, we investigated whether the expression of miRNAs could predict the clinical outcome of BC patients and therefore lead to the identification of new prognostic markers.

## Results

### MiRNA expression profiles from human breast tumors

MiRNA expression profiles were generated from 73 BC fresh-frozen samples with available mRNA profiling expression data using an optimized microarray platform for miRNA profiling. After the application of our quality control criteria, we were able to analyze 56 of the 73 samples. Patient and tumor characteristics are summarized in Table 1. In the 56 samples, on average, we detected the expression of 108 out of the 328 human miRNAs present on our arrays. The miRNAs detected in at least 90% of the samples are listed in Table S1.

**Table 1:** Summary of patient and tumor characteristics.

	Dataset	
Variable*	OXFU (untreated)	OXFT (treated)
**Sample size, No.**	73	89
**Median follow-up time, y**	9.23	7.12
**No. of relapses**	32	30
**ER status, No. (%)**		
Negative	25 (34)	
Positive	40 (55)	89 (100)
N/A	8 (11)	
**Histologic grade, No. (%)**		
1	8 (11)	18 (20)
2	27 (37)	46 (52)
3	22 (30)	17 (19)
N/A	16 (22)	8 (9)
**Lymph node status, No. (%)**		
Negative	68 (93)	50 (56)
Positive	4 (5)	34 (38)
N/A	1 (1)	5 (6)
**Tumor size, No. (%)**		
≤2 cm	43 (59)	34 (38)
>2 cm	29 (40)	55 (62)
N/A	1 (1)	
**Age, No. (%)**		
≤50 y	29 (40)	12 (13)
>50 y	44 (60)	77 (87)
**GG, No. (%)**		
Low (GG1)	25 (51)	61 (69)
High (GG3)	24 (49)	28 (31)
N/A	24 (49)	

*ER = estrogen receptor; N/A = not available; GG = genomic grade.

### Breast cancer molecular classification according to miRNA and mRNA profiling

It has repeatedly been shown that based on mRNA profiling breast tumors can be classified into four stable molecular subtypes, with the most prominent discriminators being estrogen receptor (ER), HER2 and tumor differentiation.

To assess whether miRNAs could recapitulate or even improve the previously reported molecular classification, we performed a hierarchical cluster analysis of breast tumors using both miRNAs and their corresponding protein-coding gene expression profiles. This analysis was performed in 39 out of the 56 tumor samples for which both gene expression data of protein-coding genes and miRNAs were available.

**Figure 1:**
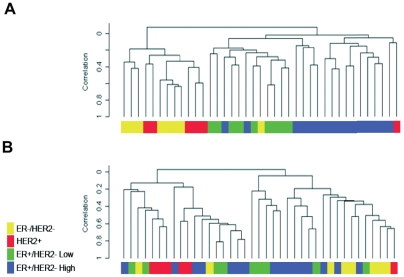
Unsupervised hierarchical clustering analysis using protein-coding genes expression profiles (A) and miRNA expression (B). Molecular subtypes were defined as described in Desmedt et al. and Wirapati et al. [16], [17] according to ER, HER2 and proliferation status. ER−/HER2− tumors are highlighted in yellow, HER2+ in red, ER+/HER2−/low proliferation (luminal A) in green and ER+/HER2−/high proliferation (luminal B) in blue.

As previously described, protein-coding gene expression profiles could segregate tumors according to the reported molecular subtypes [16],[17]. Indeed, Figure 1A shows that tumor samples could be clustered into four main subgroups, predominantly associated with ER and HER2 status. In contrast, we observed only a poor concordance with the clustering of the same tumors based on miRNAs expressions (Figure 1B). Despite the small sample size, these results suggest that miRNAs may potentially add information that is complementary to what is generated by mRNA profiling.

### Identification of miRNAs associated with clinical and pathological characteristics

We then assessed whether relevant clinico-pathological parameters such as ER status, tumor size, age and tumor grade, known to affect the clinical behavior of BC, were associated with differential miRNA expression profiles. It should be noted that the association between miRNA expression profiles and nodal status could not be analyzed since our dataset included 93% node-negative patients.

Student *t* tests at the nominal p value of 0.05 identified 19 and 20 miRNAs that could segregate breast tumors according to ER status and tumor grade respectively. These differentially expressed miRNAs are listed in Table 2. Interestingly, we found only few significant miRNAs discriminating breast tumors based on tumor size and age at diagnosis. These results suggest that in addition of being associated with distinct mRNA expression profiles [11], ER status and tumor grade are also associated with very distinct miRNA profiles, highlighting the importance of ER and tumor grade in BC biology.

**Table 2:** Differentially expressed miRNAs associated with breast cancer estrogen receptor (ER) (A) and histological grade (HG) (B) parameters.

A	Median expression			
No. samples	20	32		
Feature	ER−	ER+	p value	FDR
hsa_miR_224	3.1	1.1	0.001	0.047
hsa_miR_342	76.8	143.1	0.001	0.047
hsa_miR_186	2.1	1.2	0.003	0.128
hsa_let_7c	737.4	1073.4	0.006	0.164
hsa_miR_362	1.4	1	0.006	0.164
hsa_miR_146a	23.3	6.8	0.009	0.215
hsa_miR_345	1.5	1.1	0.011	0.218
hsa_miR_491	5.1	2.3	0.016	0.266
hsa_let_7b	641.5	934.4	0.017	0.266
hsa_let_7a	678.8	948.9	0.024	0.34
hsa_miR_20b	8.1	3.1	0.029	0.34
hsa_miR_181b	12.2	5.2	0.031	0.34
hsa_miR_130b	4.4	2.2	0.033	0.34
hsa_miR_452	1.7	1.1	0.035	0.34
hsa_miR_429	2.1	1.3	0.038	0.34
hsa_miR_181d	3.5	1.8	0.041	0.34
hsa_miR_155	7.9	3.3	0.042	0.34
hsa_miR_148a	22.5	10.5	0.043	0.34
hsa_miR_92	10.4	4.8	0.047	0.351

B	Median expression			
No. samples	7	17		
Feature	HG1	HG3	p value	FDR
hsa_miR_181b	3.5	19.9	0.001	0.064
hsa_miR_130b	1.4	6.3	0.001	0.064
hsa_miR_146a	4.8	32.4	0.002	0.083
hsa_miR_146b	11.4	40.3	0.002	0.083
hsa_miR_93	27.3	59.3	0.006	0.124
hsa_miR_181a	4.3	19	0.006	0.124
hsa_miR_20b	1.5	10.9	0.006	0.124
hsa_miR_423	1.4	4.4	0.008	0.144
hsa_miR_25	11.1	34.9	0.01	0.152
hsa_miR_106b	3.1	16	0.013	0.173
hsa_miR_186	1	2.4	0.013	0.173
hsa_miR_148a	7.4	25.1	0.017	0.206
hsa_miR_181d	1.5	4.7	0.02	0.215
hsa_miR_128a	1	2	0.032	0.32
hsa_miR_34a	11.5	25.1	0.035	0.32
hsa_miR_363	1	2.4	0.037	0.32
hsa_miR_130a	1.9	5.6	0.039	0.32
hsa_miR_345	1	1.8	0.041	0.32
hsa_miR_362	1	1.7	0.045	0.337
hsa_miR_22	83.3	154.9	0.048	0.338

FDR: False Discovery Rate.

### Identification of miRNAs associated with tumor proliferation and differentiation according to GGI

Given that gene expression grade index (GGI), reflecting tumor proliferation and differentiation, provides better biological and clinical information than histological grade [13], we investigated whether there are miRNAs differentially expressed between high and low GGI tumors, and whether these miRNAs are potential regulators of the genes composing the gene expression grade signature.

For this purpose, we contrasted miRNA expression profiles of low and high GGI tumors. This analysis identified 25 miRNAs, which were differentially expressed between the two subgroups (Table 3). As expected, 10 of the 25 identified miRNAs were also associated with histological grade. One of the differentially expressed miRNAs was the miR-210 (fold change: 3.43, p = 0.009), a miRNA that was recently reported to be associated with hypoxia [18] and with poor clinical outcome in breast cancer [19].

**Table 3:** Differentially expressed miRNAs associated with the genomic grade index (GGI).

	Median expression			
No. samples	17	22		
Feature	GG Low	GG High	p value	FDR
hsa_miR_185	2	13.4	1.10E-06	0.0001
hsa_miR_221	6.7	33	8.60E-06	0.001
hsa_miR_491	1.6	7.7	3.40E-05	0.002
hsa_miR_422b	1.7	9.4	5.09E-05	0.002
hsa_miR_146a	4.3	30.2	8.66E-05	0.002
hsa_miR_222	1.7	6.4	0.0004	0.01
hsa_miR_181b	3.3	14.6	0.001	0.015
hsa_miR_130b	1.6	5.7	0.001	0.015
hsa_miR_146b	10.4	37.1	0.001	0.019
hsa_let_7i	83	143.5	0.001	0.02
hsa_miR_22	61.7	173.2	0.002	0.023
hsa_miR_155	2.4	9.2	0.004	0.043
hsa_miR_223	2.2	8	0.004	0.043
hsa_miR_379	2.3	6.9	0.004	0.043
hsa_miR_224	1	3.2	0.006	0.059
hsa_miR_210	2.3	7.9	0.009	0.077
hsa_miR_423	1.5	3.6	0.011	0.089
hsa_miR_181a	4.9	13.6	0.011	0.089
hsa_miR_362	1	1.6	0.025	0.184
hsa_miR_346	1.1	1.8	0.027	0.191
hsa_miR_432	1.6	3.4	0.032	0.218
hsa_miR_151	2	4.3	0.034	0.218
hsa_miR_186	1.2	2	0.044	0.264
hsa_miR_191	52.4	109.3	0.046	0.264
hsa_miR_422a	1.4	2.9	0.046	0.264

FDR: False Discovery Rate.

### MiRNAs associated with clinical outcome

MiRNAs potential prognostic value was investigated by contrasting the expression levels of 328 human miRNAs with clinical outcome using our systemically untreated BC series with a median follow-up of 9,2 years. This analysis identified 2 human miRNAs (miR-210 and miR-148a) that were significantly associated with relapse-free survival (RFS) at a p value<0.05. Given its association with the GGI on one hand and its association with RFS on the other hand, we decided to concentrate on miR-210.

We first aimed to confirm the differential expression level of miR-210 between high and low GGI tumors using quantitative real-time PCR (qRT-PCR). All tumor samples showed detectable expression levels of miR-210 using qRT-PCR. This contrasted with only 61% when assessed by microarrays, demonstrating the higher detection sensitivity of the qRT-PCR technique. Despite this difference in the detection level between the two techniques, we found a statistically significant correlation in miR-210 expression between the two assays (Spearman ρ = 0.7, p = 10^−6^). Furthermore, qRT-PCR analysis confirmed the statistically significantly differential expression levels of miR-210 between low and high GGI tumors. High GGI tumors were associated with 2.73 times higher expression levels compared to low GGI tumors (p = 6.10^−4^).

Next, in order to further investigate the association between the GGI and miR-210 targets, we performed a gene set enrichment analysis using the microarray data used in the original publication [13]. The resulting p-value computed on 1000 random permutations was highly significant (0.002), confirming the association between GGI-low/high subgroups and mir-210 targets. Although we did not find any gene in common between the miR-210 target genes and the GGI genes, we observed, when investigating the networks of these target genes together with the GGI genes with Ingenuity Pathway Analysis, that the 3 networks representing cell cycle genes involved a mixture of miR-210 target genes and GGI genes (Figures S1, S2 and S3).

The fact that high expression levels of miR-210 were associated with a higher risk of recurrence (p = 0.035) concur with recent findings reported by Camps et al. [19]. We then aimed to confirm the prognostic value of miR-210 by qRT-PCR. When considered as a continuous variable in a univariate analysis, miR-210 expression was statistically significantly associated with RFS (p = 2.10^−4^). Interestingly, when performing subgroup analyses based on ER status, miR-210 was statistically associated with RFS, both in the ER-positive (n = 32, p = 0.004) and the ER-negative populations (n = 20, p = 0.008).

**Figure 2:**
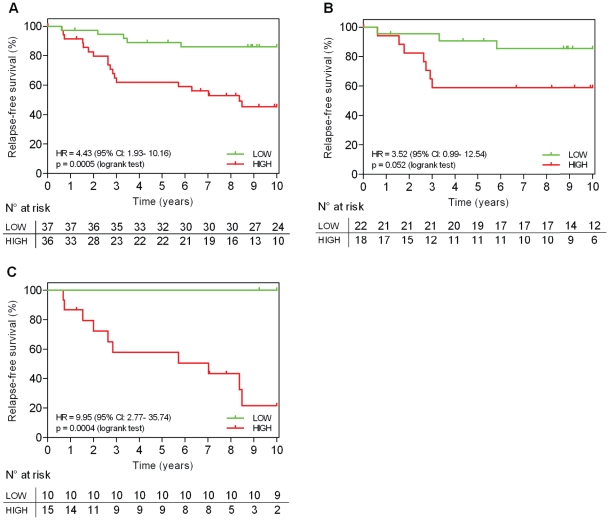
Kaplan-Meier analyses for relapse-free survival according to miR-210 expression levels. (A) Systemically untreated population (N = 73 OXFU); (B) untreated ER-positive population (N = 40); (C) untreated ER-negative population (N = 25). HR: hazard ratio, CI: confidence intervals.

Similar results were found in a Kaplan-Meier analysis, for which patients (N = 73) were categorized on the basis of miR-210 median expression value. A statistically significant association was observed between a high miR-210 expression level (above the median) and a higher risk of recurrence [HR = 4.43 (95% CI: 1.93–10.16), p = 5.10^−4^] (Figure 2A). The Kaplan-Meier curves for the ER-positive [HR = 3.52 (95% CI: 0.99–12.54); p = 0.052] and ER-negative [HR = 9.95 (95% CI: 2.77–35.74), p = 4.10^−4^] populations are illustrated in Figures 2B and 2C, with 55% (22/40) and 40% (10/25) of the patients assigned to the low-risk group respectively. MiR-210 thus appears to be associated with worse clinical outcome both in ER-positive and ER-negative BC.

### MiR-210 expression separates histological grade 2 breast tumors into two groups with distinct clinical outcomes

**Figure 3:**
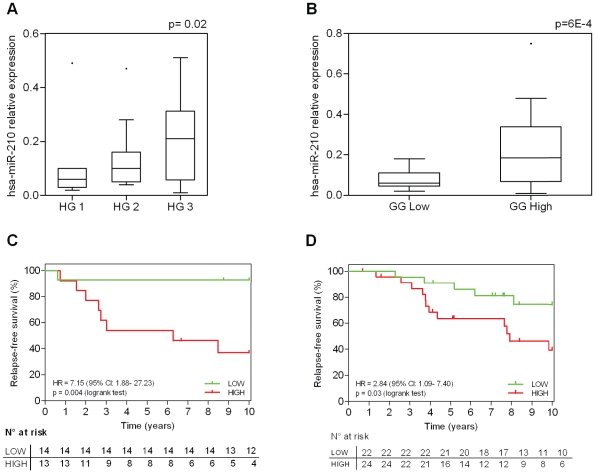
MiR-210 relative expression according to histological grades and genomic grades. The normalized expression levels of miR-210 between histological grades (HG) 1, 2 and 3 (A) and between low and high GGI (GG Low and GG High) (B) assessed by qRT-PCR. Boxes represent interquartile range, black bars indicate the median, points represent outliers. p value is based on one-way Anova test. Kaplan-Meier analyses for relapse-free survival according to miR-210 expression levels in histological grade 2 population. (C) Systemically untreated population (N = 27); (D) Validation ER-positive tamoxifen treated population (N = 46) HR: hazard ratio, CI: confidence intervals.

One of the most interesting findings of the GGI was its ability to identify two clinically relevant subgroups within the histological grade 2 tumors. In the current study, we investigated whether miR-210 expression levels could recapitulate the prognostic ability of GGI, particularly within the histological grade 2 subgroup. The normalized expression levels of miR-210 between histological grades (HG) 1, 2 and 3 and between low and high GGI (GG Low and GG High) assessed by qRT-PCR are shown in Figure 3A and 3B respectively. As expected, grade 2 tumors spanned the expression levels of miR-210 of grade 1 and grade 3 tumors. Like GGI, the expression levels of miR-210 divided grade 2 tumors into two subgroups with distinct clinical outcomes. Grade 2 tumors with higher expression levels of miR-210 (above the median) were associated with worse RFS [HR = 7.15 (95% CI: 1.88–27.2), p = 0.004] (Figure 3C), demonstrating that miR-210 recapitulates the prognostic information of GGI in a similar manner.

As the size of the dataset is small, we aimed to confirm these results on an independent dataset of 89 ER-positive breast cancer patients homogeneously treated in the adjuvant setting with tamoxifen for 5 years and with long follow-up. As in the first dataset, grade 2 tumors could be separated in two groups of distinct clinical outcome according to miR-210 expression levels, a high level of miR-210 being associated with worse RFS [HR = 2.84 (95% CI: 1.09–7.40), p = 0.03] (figure 3D), confirming that the prognostic information of GGI is recapitulated by miR-210 in a similar manner.

### MiR-210 as a prognostic marker in ER-positive tamoxifen only treated breast cancer patients

**Figure 4:**
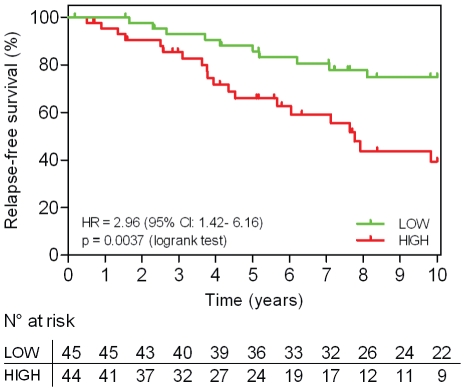
Kaplan-Meier analyses for relapse-free survival according to miR-210 expression levels. Validation ER-positive tamoxifen treated population (N = 89) HR: hazard ratio, CI: confidence intervals.

Since from our previous work [14] high GGI was associated with high risk of recurrence after adjuvant tamoxifen therapy in ER-positive patients, we sought to investigate whether the expression of miR-210 could also identify two ER-positive subgroups with distinct clinical outcomes. For this purpose the expression of miR-210 was evaluated in the second cohort of 89 ER-positive tamoxifen only treated breast cancer patients (Table 1). We applied similar cut-off to the one defined on the untreated population in order to separate the population into two groups, assigning 51% (45/89) of the patients to the low risk group. As observed in the initial dataset, a statistically significant survival difference was observed between the two groups [HR = 2.96 (95% CI: 1.42–6.16), p = 0.004], a high level of miR-210 expression being associated with a higher risk of recurrence than a lower level of miR-210 (Figure 4). MiR-210 remained significant in a multivariate analysis [HR = 4.4 (95% CI: 1.65–11.76, p = 0.003], together with patient age [HR = 4.74 (95% CI: 1.83–12.35, p = 0.001] and tumor size [HR = 3.53 (95% CI: 1.28–9.78, p = 0.015]. The histological grade, the GGI and the nodal status were included in the multivariate analysis and have been substituted by miR-210. Therefore, miR-210 is associated with poor clinical outcome under tamoxifen treatment.

### Comparison of the prognostic accuracy of miR-210 and recently published prognostic gene expression signatures

Since proliferation and differentiation, captured by the genomic grade, seem to be the common denominator for the prognostic performance of several multi-gene signatures, we assessed whether miR-210 alone could provide similar information [17].

**Figure 5:**
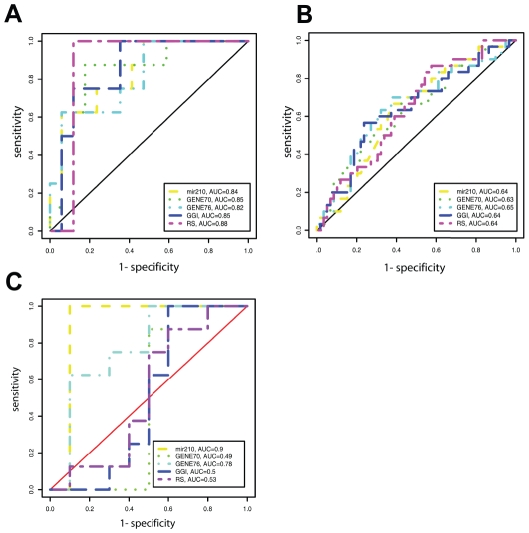
MiR-210 shows similar prognostic performance to multiple genes signatures. Time-dependent receiver operating characteristic curves for miR-210 expression, the gene expression grade index (GGI), the 70-genes signature (GENE70), the 76-genes signature (GENE76) and the estimated 21-gene recurrence score (ONCOTYPE) for relapse-free survival. (A) Systemically untreated ER-positive population (N = 25); (B) ER-positive tamoxifen-treated population (N = 89) and (C) ER-negative untreated population (N = 18). Areas under the curves are shown beneath the curves.

To address this, we computed time-dependent receiver operating characteristic curves (ROC) for miR-210 expression level and several prognostic signatures – the gene expression grade index (GGI) [13], the 70-gene signature (GENE70) [20], the 76-gene signature (GENE76) [12] and the estimated 21-gene recurrence score (RS) [21] - for RFS within 10 years in the ER-positive patients in both the training dataset and the tamoxifen-treated breast cancer dataset (Figures 5A and 5B). Gene signature computation is detailed in material and methods section.

The areas under the ROC curves demonstrate that miR-210 shows similar prognostic performance to the multiple genes signatures evaluated in this study. Of interest, similar results were found in an exploratory analysis in the ER-negative subpopulation of the initial dataset (Figure 5C). However, these results need further validation.

### Involvement of miR-210 in different biological processes

As miR-210 expression is correlated to poor prognosis both in ER-positive and in ER-negative BC patients, we aimed to investigate the biological processes regulated by miR-210 and which may elucidate its function in the aggressive phenotype of high grade BC. We performed in silico functional analyses of the genes deregulated upon miR-210 overexpression and repression in two different BC cell lines. MCF7 cells were used as a model for low grade ER-positive BC as they are low-proliferating ER-positive cells which express miR-210 at a level comparable to low grade tumours according to qRT-PCR (Figure S4). We overexpressed miR-210 (or a control sequence) in MCF7 cells (MCF7-miR-210 and MCF7-control respectively) using lentiviral transduction and miR-210 overexpression was confirmed by qRT-PCR. Gene expression profiling analysis of these cells revealed the upregulation of 897 genes and downregulation of 922 genes upon miR-210 overexpression (fold change >2, Table S2). As a model to investigate the role of miR-210 in ER-negative BC, we used the ER-negative highly-proliferating MDA-MB-231 cell line which expresses miR-210 at a high level as high grade tumours (Figure S4). We repressed miR-210 in MDA-MB-231 cells by overexpressing a sequence complementary to miR-210 (or a control sequence) using lentiviral transduction and miR-210 repression was confirmed by qRT-PCR. Gene expression profiling analysis of these cells revealed that repression of miR-210 in MDA-MB-231 cells led to the upregulation of 28 genes and downregulation of 58 genes (fold change >2, Table S3). Interestingly, gene set enrichment analyses investigating Gene Ontology (GO) categories of the differentially expressed genes showed that GO categories/pathways such as cell adhesion, extracellular structure organization, epithelial cell proliferation, cell division, cell cycle and immune response were significantly over-represented (p<0.05) (Tables 4A and B).

**Table 4:** Examples of significantly impacted biological pathways in MCF7 cells upon miR-210 overexpression (A) and in MDA-MB-231 cells upon miR-210 repression (B).

A		
GO category	Description	LS permutation p value
GO:0007067	mitosis	0.00001
GO:0051301	cell division	0.00015
GO:0043281	regulation of caspase activity	0.0002
GO:0006916	anti-apoptosis	0.00279
GO:0002418	immune response to tumor cell	0.00501
GO:0016337	cell-cell adhesion	0.00592
GO:0030054	cell junction	0.01132
GO:0051726	regulation of cell cycle	0.01235
GO:0045596	negative regulation of cell differentiation	0.03377
GO:0007346	regulation of mitotic cell cycle	0.04383
GO:0007160	cell-matrix adhesion	0.04499
GO:0007093	mitotic cell cycle checkpoint	0.04942

B		
GO category	Description	LS permutation
p value		
GO:0002684	regulation of immune system	0.00001
GO:0016337	cell-cell adhesion	0.00001
GO:0005911	cell-cell junction	0.00001
GO:0050776	regulation of immune response	0.00002
GO:0043062	extracellular structure organization	0.0006
GO:0005201	extracellular matrix structural constituent	0.00067
GO:0000904	cell morphogenesis involved in differentiation	0.00452
GO:0007626	locomotion	0.01268
GO:0007015	actin filament organization	0.01885
GO:0050673	epithelial cell proliferation	0.04524

### MiR210 is involved in cell proliferation both in ER-positive and ER-negative breast cancer cell lines

**Figure 6:**
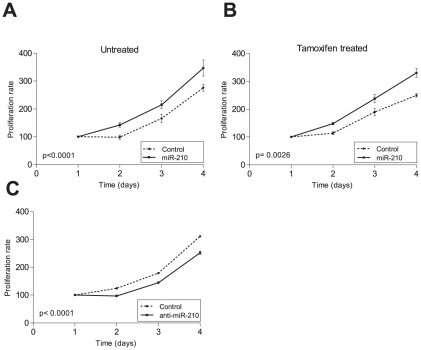
Involvement of miR-210 in cell proliferation. Cell growth curves for MCF7-miR-210 and MCF7-control in untreated (A) and Tamoxifen-treated (10^−7^ M) conditions (B). Cell growth curves for MDA-MB-231-anti-miR-210 and MDA-MB-231-control (C).

As gene set expression comparisons revealed that genes involved in cell cycle and proliferation were deregulated upon miR-210 overexpression in MCF7 cells and miR-210 repression in MDA-MB-231 cells, we aimed to investigate miR-210 role in the proliferative phenotype of high grade tumours. Cell proliferation assays revealed that miR-210 overexpression led to a statistically significant increase in MCF7 cell proliferation (MCF7-miR-210) compared to the control cells (MCF7-control) both in untreated and in Tamoxifen treated cells according to a two-way Anova analysis (Figures 6A and 6B). Similarly, MDA-MB-231 proliferation rate decreased upon miR-210 downregulation (MDA-MB-231-anti-miR-210) compared to the control cells (MDA-MB-231-anti-miR-control) (Figure 6C). In conclusion, the association between miR-210 expression and clinical outcome could rely on its role as a tumor proliferation regulator both in ER-positive and ER-negative breast cancer patients.

### Role of miR210 in cell migration and invasion

**Figure 7:**
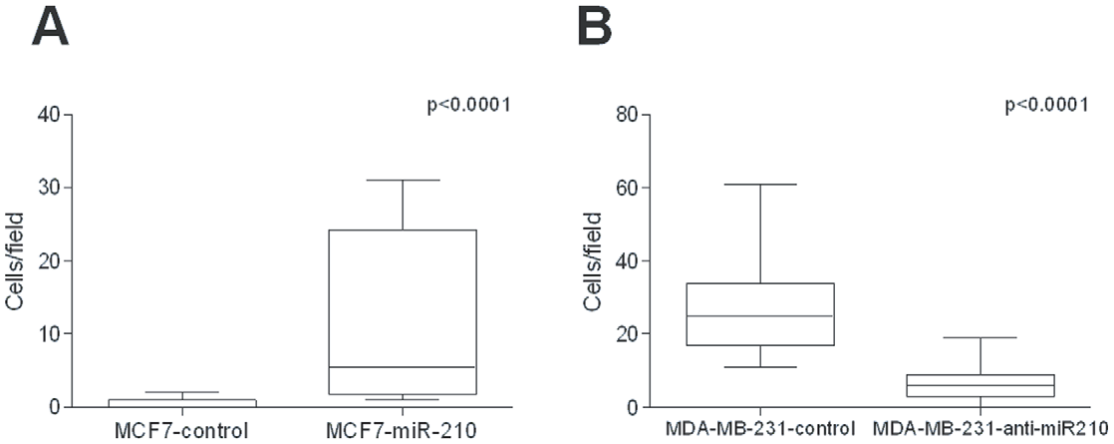
MiR-210 involvement in cell invasion and migration. MiR-210 overexpression enhances MCF7 cell invasion. Illustration of the number of invading cells upon miR-210 overexpression compared to the control cells (A). MiR-210 repression enhances MDA-MB-231 cell migration. Illustration of the number of migrating cells upon miR-210 repression compared to the control cells (B).

To further define miR-210 role in the aggressive phenotype of high grade tumors and as genes involved in processes such as cell adhesion, extracellular matrix structure, extracellular organization are deregulated upon miR-210 overexpression in MCF7 cells and miR-210 repression in MDA-MB-231 cells, we aimed to investigate miR-210 role in cell migration and invasion in the lentiviral transduced cell lines previously used. As shown in Figure 7A, miR-210 overexpression in non-invading MCF7 cells led to cell invasion in a chamber invasion assay. MiR-210 repression in migrating and invading MDA-MB-231 cells led to a decrease in cell migration compared to the control cells (Figure 7B). Figure S5 illustrates representative fields of cells which have acquired migration and invasion capabilities after miR treatment compared to the control cells. These results suggest that miR-210 prognostic potential could rely on its role as a cell invasion regulator in ER-positive BC patients and as a regulator of cell migration in ER-negative breast cancer patients.

## Discussion

We optimized a microarray platform which allowed us to analyze the profiles of the miRNAs expressed in primary human BC samples from systemically untreated patients. MiRNA expression profile has been investigated in human BC in two previous studies comparing tumor samples to normal tissue [3], [22]. Here we focused on miRNA profiling on tumor samples only. On average, we detected 108 out of the 328 human miRNAs present on our arrays. This falls in the same range as the Blenkiron et al. study which detected 133 miRNAs in BC samples while analyzing the miRNAs associated with the BC molecular subtypes using a bead-based method [23].

Through our integrated analysis of combined miRNA and protein-coding gene profiling, and regarding the sample size, our results suggest that the molecular subclasses identified by mRNA profiling (basal/luminal and HER2/Neu) could not be accurately reproduced by clustering the miRNA profiles. This suggests that miRNAs might provide additional and complementary information regarding BC molecular classification. Interestingly, Blower et al. came to the same conclusion when comparing the NCI-60 cancer cell line groupings based on miRNAs and mRNAs expression profiles [24].

In this study, several miRNAs associated with different clinico-pathologic characteristics were identified. In particular, 19 miRNAs that could segregate ER+ and ER− breast tumors were identified. Specifically, within this list, we identified 6 and 5 miRNAs which were also recently shown to be associated with ER status in the studies published by Blenkirion et al. [23] (miR-342, let-7a, let-7b, let-7c overexpressed in ER+ tumors, and miR-148a and miR-155) and Janssen et al. (miR146a, miR-155, miR-224, miR-362, and miR-155) [25]. We further identified 20 miRNAs that can distinguish high and low histological grade breast tumors. Three of them (miR-106b, miR25 and miR-93) were also found to be associated with the histological grade, as identified by Blenkiron et al. [23]. These 3 miRNAs were also shown by Janssen et al. to be associated with the mitotic index and they showed that miR-106b was the miRNA, which correlated the strongest with proliferation [25]. Interestingly, Van der Auwera et al. also reported significant associations between ER status and tumor grade, but not for tumor size, nodal status, tumor stage or HER2 status [26]. Altogether, our findings reinforce the repeated observation that, at diagnosis, ER status and tumor grade are associated with specific miRNA expression profiles, whereas this is not the case for tumor size and age.

Since our group recently reported that proliferation and differentiation, captured by GGI, seem to be the main biological processes associated with BC prognosis and form the common denominator of different prognostic signatures, we investigated whether the genes composing the GGI were regulated at the miRNA level. We identified 25 miRNAs that were differentially expressed between low and high gene expression grade tumors.

Although, several miRNAs would have been potentially interesting for further investigation, we decided to concentrate on miR-210 because it was the only miRNA which in addition to being associated with the GGI, was also associated with prognosis in our dataset, which is consistent with the recently reported results by Camps et al. [19].

Interestingly, this miRNA was also shown to be upregulated in BC cell lines in hypoxic conditions, a key feature of the tumor microenvironment. Overexpression of miR-210 has also been reported in various tumors, including breast cancer [3], [22]. Furthermore, it has been shown to decrease pro-apoptotic signaling in a hypoxic environment, suggesting an impact of this miRNA on tumor formation [27].

The fact that some inconsistencies exist between different miRNA profiling analyses studies is most likely due to technical and analytical variations, highlighting the need for standardization. Regardless of the technology used, confirmation with another analytical approach is critical for the validation of findings. We therefore decided to first validate our findings on the miR-210 using qRT-PCR and found a strong correlation between the microarray and qRT-PCR results.

Second, we confirmed the association of the GGI and miR-210 target genes using a gene set enrichment analysis. Also, an analysis of the literature of the miR-210 target genes revealed that repression of those genes in high grade tumors could potentially be responsible for the proliferative phenotype of these tumors. For example, ACVR1B (activin receptor 1B), which is a member of the transforming growth factor beta superfamily that regulates mammary cell function during development, lactation, and in cancer has been shown to slow down the growth of breast cancer cells by inducing G(0)/G(1) cell cycle arrest [28]. Also, CBFA2T1, also referred to as MTG16, has been shown to have a growth inhibiting role in breast cancer cell lines [29]. Additionally, recent evidence indicates that the expression of DICER1, which is the key enzyme required for the biogenesis of microRNAs and small interfering RNAs and is essential for both mammalian development and cell differentiation is associated with hormone receptor status and cancer subtype in breast tumours and that its downregulation may be related to the metastatic spread of tumours [30].

Third, we further investigated the prognostic value of miR-210, for which high expression levels of miR-210 were associated with a higher risk of recurrence. The prognostic power of miR-210 on the initial dataset was confirmed using qRT-PCR. We found that this miRNA was associated with shorter RFS on 40 ER-positive BC patients. We further demonstrated that this miRNA predicted poor clinical outcome in an independent dataset of 89 tamoxifen-only treated patients with ER-positive early BC. Intriguingly, we showed that this single miRNA had similar prognostic performance with the previously published BC multi-gene signatures. The above multi-gene signatures are mainly informative in ER-positive rather than ER-negative tumors [17]. The only two multi-gene signatures reported to carry prognostic information in the ER-negative subgroup were composed of genes related to immune response [16], [31]. Interestingly, miR-210, apart from being informative in ER-positive disease, also identified two subgroups with distinct clinical outcome in patients with ER-negative BC. However, given the small number of patients, further validation of this finding on an independent dataset is needed.

We next performed functional analyses to investigate the association of miR-210 expression and disease outcome. MiR-210 overexpression in MCF7 cells and repression in MDA-MB-231 cells induced the deregulation of genes involved in different biological processes such as cell cycle, cell adhesion and immune response. Cell proliferation assays revealed that miR-210 overexpression enhances MCF7 cell proliferation suggesting that the prognostic potential of miR-210 may rely on its role in tumor proliferation both in untreated and tamoxifen-only treated ER-positive BC. Similarly, MDA-MB-231 cell growth decreased upon miR-210 repression suggesting that the association between miR-210 and clinical outcome in ER-negative BC may also rely on its role as a cell proliferation regulator. Migration and invasion assays suggested that miR-210 prognostic potential could also rely on its role as a cell invasion regulator in ER-positive BC patients and as a regulator of cell migration in ER-negative breast cancer patients.

Recent findings demonstrated that e2f transcription factor 3, a key cell cycle protein, was regulated by miR-210 and that high frequency of miR-210 gene copy deletions were found in ovarian cancer patients [18]. Using the same cell lines model, we showed that miR-210 is involved in the regulation of cell migration and invasion potentially explaining partially the aggressive phenotype of high grade tumors. The association of miR-210 expression and disease outcome could also be due to its anti-apoptotic action that has been recently identified in cell lines or to its implication in hypoxia response.

In conclusion, miRNA and mRNA profiling seem to be complementary tools that can improve our understanding of BC biology. A single miRNA (miR-210), quantified using a simple and accurate qRT-PCR assay, showed prognostic performance that was similar to that of various multi-gene signatures. These results need further prospective validation.

## Materials and Methods

### Patient samples

Tissue samples used in this study were provided from the John Radcliffe Hospital, Oxford, UK. The first population included fresh-frozen primary tumor samples from 73 BC patients who had not received any adjuvant systemic treatment. This population is referred to as OXFU. Protein-coding gene expression profiles, using the Affymetrix U133A Genechips (Affymetrix, Santa Clara, CA), were available for 49 of the 73 patients (raw data are available at the Gene Expression Omnibus (GEO) repository database [http://www.ncbi.nlm.nih.gov/geo/], accession number GSE2990) [13].

The second dataset, referred as OXFT, consisted of fresh-frozen primary tumor samples from 89 ER-positive BC patients homogeneously treated with tamoxifen only. Protein-coding gene expression profiles were also available for all patients (GSE6532) [14].

Table 1 summarizes patient and tumor characteristics from both populations. Relapse-free survival (RFS) was defined as the interval between the dates of breast surgery and diagnosis of any type of relapse (local, regional or distant).

### MiRNA microarray hybridization and data normalization

Total RNA from breast tumor samples was isolated using Trizol method (Invitrogen, Carlsbad, CA). MiRNA labeling and hybridization on mirVana miRNA Bioarrays V2 were performed according to the manufacturer's recommendations using 7.5 µg of total RNA and the mirVana miRNA labeling kit (Ambion, Austin, TX). The Bioarrays used for miRNAs expression profiling comprise a total of 640 probes targeting 328 human miRNAs (hsa-miRs), as well as mouse and rat miRNAs, from the miRBase Sequence Database version 8.0. (http://microrna.sanger.ac.uk/) [32], [33]. Samples with artifacts such as non specific hybridization or high background intensities have been excluded from analysis (quality control criteria). Hybridization signals were detected at 532 nm using a GenePix 4000B scanner and quantified by GenePix Pro 4.0 software (Axon Instruments, Downingtown, PA). The signal intensity of each spot was calculated by subtracting the local background from the mean signal. After averaging replicate spots, a global scaling normalization was applied, as suggested by Ambion. This normalization consisted of the following steps: 1) thresholding, for which intensities below the background (set to 2 times the signal of empty spots) were set to 1; 2) scaling, which involved ranking the intensities of each sample separately and dividing them by the 30^th^ intensity value; and 3) log 2 transformation. The normalized intensities were used for further analyses. Data are available at the Gene Expression Omnibus (GEO) repository database [http://www.ncbi.nlm.nih.gov/geo/], accession number GSE28321).

### Quantitative real-time PCR (qRT-PCR)

The analysis of miR-210 expression by quantitative real-time PCR was performed according to the TaqMan MicroRNA Assay protocol (Applied Biosystems, Foster City, CA). Briefly, 10 ng of total RNA were reverse-transcribed using the MicroRNA Reverse Transcription kit and a specific stem-loop primer according to the manufacturer's instructions (Applied Biosystems, Foster City, CA). Real-time PCR was performed on a 7900HT Sequence Detection System (Applied Biosystems, Foster City, CA). All reactions were run in triplicate. MiR-210 expression relative to small nucleolar RNAs RNU44 and RNU48 was calculated using the 2^−ΔCt^ method. This normalized expression level allowed us to determine the fold changes in miR-210 expression between tumor subgroups.

### Cell culture

MCF7 and MDA-MB-231 cells were obtained from the ATCC and cultured under standard conditions. MiR-210-overexpressing MCF7 cells (MCF7-miR-210) and MCF7 control cells (MCF7-control) were cultured under the same conditions as the parental MCF7 cells. MDA-MB-231 cells in which miR-210 was downregulated (MDA-MB-231-anti-miR-210) and the corresponding control (MDA-MB-231-control) were cultured under the same conditions as the parental MDA-MB-231 cells.

### Lentiviral production and transduction

Lentiviral vector production was performed as previously described [34]. Briefly, vesicular stomatitis virus glycoprotein G (VSV-G) pseudo-typed lentiviral particles were generated by polyethylene imine (Sigma, St. Louis, MO) co-transfection of 293T cells with three plasmids pMIRNA, pCMVDR8.91 and pMD.G. pCMVDR8.91 is HIV-derived packaging construct that encodes the HIV-1 Gag and Pol precursors as well as the regulatory proteins Tat and Rev. Vesicular stomatitis virus glycoprotein G (VSV-G) was expressed from pMD.G. pMIRNA (System Biosciences) is a lentiviral-based vector in which miR-210 precursor sequence (for miR-210 overexpression), a sequence complementary to miR-210 (for miR-210 repression) or a scramble sequence (negative control) have been cloned downstream of the CMV promoter. This vector contains copepod GFP as a reporter gene allowing GFP-positive cells sorting by flow cytometry. Twenty-four 24 h after transient transfection of 293 T cells, viral supernatants were collected, filtered and concentrated. For viral transduction, MCF7 and MDA-MB-231 cells were plated at a density of 10^5^ cells/well in 12-wells culture plates and exposed to lentiviral preparations with MOI 5 in a volume of 500 ml in the presence of 8 mg/ml polybrene. GFP-positive cells were sorted by flow cytometry at day 7 after transduction.

### Cell proliferation assay

Cell proliferation was determined by 3-(4,5-dimethylthiazole-2-yl)-2.5 diphenyltetrazolium bromide assay (MTT, Sigma). MCF7-miR-210 and MCF7-control cells were seeded at a density of 1500 cells per well. MDA-MB-231-anti-miR-210 and MDA-MB-231-control cells were seeded at a density of 2000 cells per well. At each time point, 100 µl of 5 mg/ml MTT was added and incubated at 37°C for 3 h and 100 µl DMSO was added to the wells. Every 24 hours, the rate of cellular proliferation was determined by measuring the absorbance at 590 nm. Cell growth curves were calculated as mean values of six replicates after normalization to the absorbance at day 1. Difference in cell growth was considered as significant when p<0.05 according to a two-way Anova test.

### Cell migration and invasion

Twenty-four well migration and invasion chambers (Cell Biolabs, San Diego, CA) were used to study cell migration and invasiveness respectively according to manufacturer's instructions. Migratory and invading cells were counted under a light microscope with five individual fields per insert. Results are presented as the average of triplicate experiments. Differences in cell migration and invasion rates were assessed using a Mann Whitney test and were considered significant when p<0.05.

### Protein-coding gene expression profiling

Microarray analysis was performed using the Affymetrix U133A Genechips following manufacturer's instructions (Affymetrix, Santa Clara, CA). Probe quantification and data normalization were performed as previously [13]. Protein-coding gene expression profiles (raw data) are available at the Gene Expression Omnibus (GEO) repository database [http://www.ncbi.nlm.nih.gov/geo/], accession number GSE25162). Genes were considered as differentially expressed when the fold change between the 2 classes was >2.

### Gene Signature Computation

Because we used different gene expression profiling technology than some of the gene signatures investigated in this study (i.e. GGI [13], GENE70 [20], GENE76 [12] and Recurrence Score (RS) [21], we defined gene signature as:
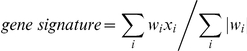
where *x_i_* is the expression of a gene in the gene signature that is present in the dataset's platform. *w_i_* is either +1 or −1 depending on the sign of the gene-specific weights (coefficients, correlations, or other measures) from the original. Robust scaling was performed on each gene signature in order to have the interquartile range equal to 1 and the median equal to 0 within each dataset, allowing for comparison between gene signatures. It is worth mentioning that we computed the low and the high gene expression grade indices as we did in our original publication [13], i.e. by defining the cutoff as the middle point between the means of gene expression grade indices for patients with histological grades 1 and 3.

### Statistical analysis

Cluster analyses were conducted to look for natural groupings in the mRNA and miRNA expression profiles. Unsupervised hierarchical clustering was performed with uncentered correlation and average linkage using BRB-ArrayTools software. Molecular subtypes were defined as described in Desmedt et al. [16] and Wirapati et al. [17]. In supervised analyses, we assessed whether clinical parameters were associated with differential miRNA expression profiles using Student *t* tests. We also compared low versus high gene expression grade tumor samples (see Section Gene Signature Computation). For these comparison analyses, we only considered miRNAs expressed in at least 20% of the samples. All p-values were two-tailed and the difference in miRNA expression was reported as significant when p<0.05. Correlation of miR-210 expression by microarray and qRT-PCR analysis was assessed using Spearman's rank test. For survival analysis, we used two accuracy measures in order to estimate the relevance of a variable for survival prediction. First, we used the traditional Cox's proportional hazards model [35] to estimate the hazard ratio of such a variable. Second, we used the time-dependent ROC curve as defined by Heagerty et al. [36] and its related area under the curve (AUC). To compare the relevance of two variables for survival predictions, we compared the hazard ratios by a paired Student *t* test. The Kaplan-Meier method was used to estimate the survival curves. Patients were separated into two groups according to miR-210 relative expression level, the median value of the initial dataset being used as the cut-off. The same threshold was used for the validation dataset in order to avoid overfitting. The survival data were censored to 10 years. The statistical significance of global gene expression changes in Gene Ontology (GO) categories was assessed by LS permutation tests. Statistical analyses were performed using BRB-ArrayTools software (available at http://linus.nci.nih.gov/BRB-ArrayTools.html), SPSS 15.0 and GraphPad Prism 5.

### Prediction of miRNA target genes

We used the miRanda algorithm for miRNA target genes prediction. (www.microRNA.org) [37], [38].

### Association of miRNA target genes

To test the association between miRNA targets and existing gene signatures, we performed a gene set enrichment analysis [39]. The class comparison performed to define the gene signature of interest was done using the gene expression data used in the original publication. The resulting full ranked list of genes was then used in combination with the list of targets genes predicted by the miRanda algorithm (gene set) to compute the enrichment Score (ES). In order to estimate the null distribution of the ES, the class labels used to define the signature from the gene expression data were randomly permutated 1000 times. If the observed ES is significantly higher that the random ES (permutated p-value<0.05) it meant that the miRNA targets are significantly associated with the gene signature, or more precisely with the biological phenomenon quantified by this signature.

We performed a gene set enrichment analysis to assess the association between the Gene expression Grade Index (GGI) and the target genes of miR-210, following the approach described above. The ES, defined as the maximum Kolmogorov-Smirnov ranking sum [39], was significantly higher than ES computed after random permutations of the labels used to define the GGI from the original gene expression data, therefore confirming the association between GGI and mir-210 targets.

Network analyses involving miR-210 target genes and GGI genes were performed using Ingenuity Pathways Analysis (IPA) tools (www.ingenuity.com), a web-delivered application that enables researchers to discover, visualize, and explore molecular interaction networks in gene expression data.

## Supporting Information

Figure S1 Gene network n°1 involving cell cycle genes from the analysis from IPA including the miR-210 target genes and GGI genes.
(TIF)

Figure S2 Gene network n°2 involving cell cycle genes from the analysis from IPA including the miR-210 target genes and GGI genes.
(TIF)

Figure S3 Gene network n°3 involving cell cycle genes from the analysis from IPA including the miR-210 target genes and GGI genes.
(TIF)

Figure S4 MiR-210 relative expression in MCF7 and MDA-MB-231 BC cell lines compared to BC samples according to histological grades (HG) and genomic grades (GG).
(TIF)

Figure S5 MiR-210 involvement in cell invasion and migration. MiR-210 overexpression enhances MCF7 cell invasion. Illustration of invading cells upon miR-210 overexpression compared to the control cells (A). MiR-210 repression enhances MDA-MB-231 cell migration. Illustration of migrating cells upon miR-210 repression compared to the control cells (B).
(TIF)

Table S1 List of the miRNAs detected in at least 90% of the breast cancer samples.
(XLSX)

Table S2 List of the genes deregulated (fold change FC>2) upon miR-210 overexpression in MCF7 cells.
(XLSX)

Table S3 List of the genes deregulated (fold change FC>2) upon miR-210 repression in MDA-MB-231 cells.
(XLSX)
