# Supplementary figures and images for: Global MicroRNA Expression Profiling Identifies MiR-210 Associated with Tumor Proliferation, Invasion and Poor Clinical Outcome in Breast Cancer
Source: PLoS One. 2011 Jun 29;6(6):e20980. doi: 10.1371/journal.pone.0020980 (PMC3126805; doi:10.1371/journal.pone.0020980)

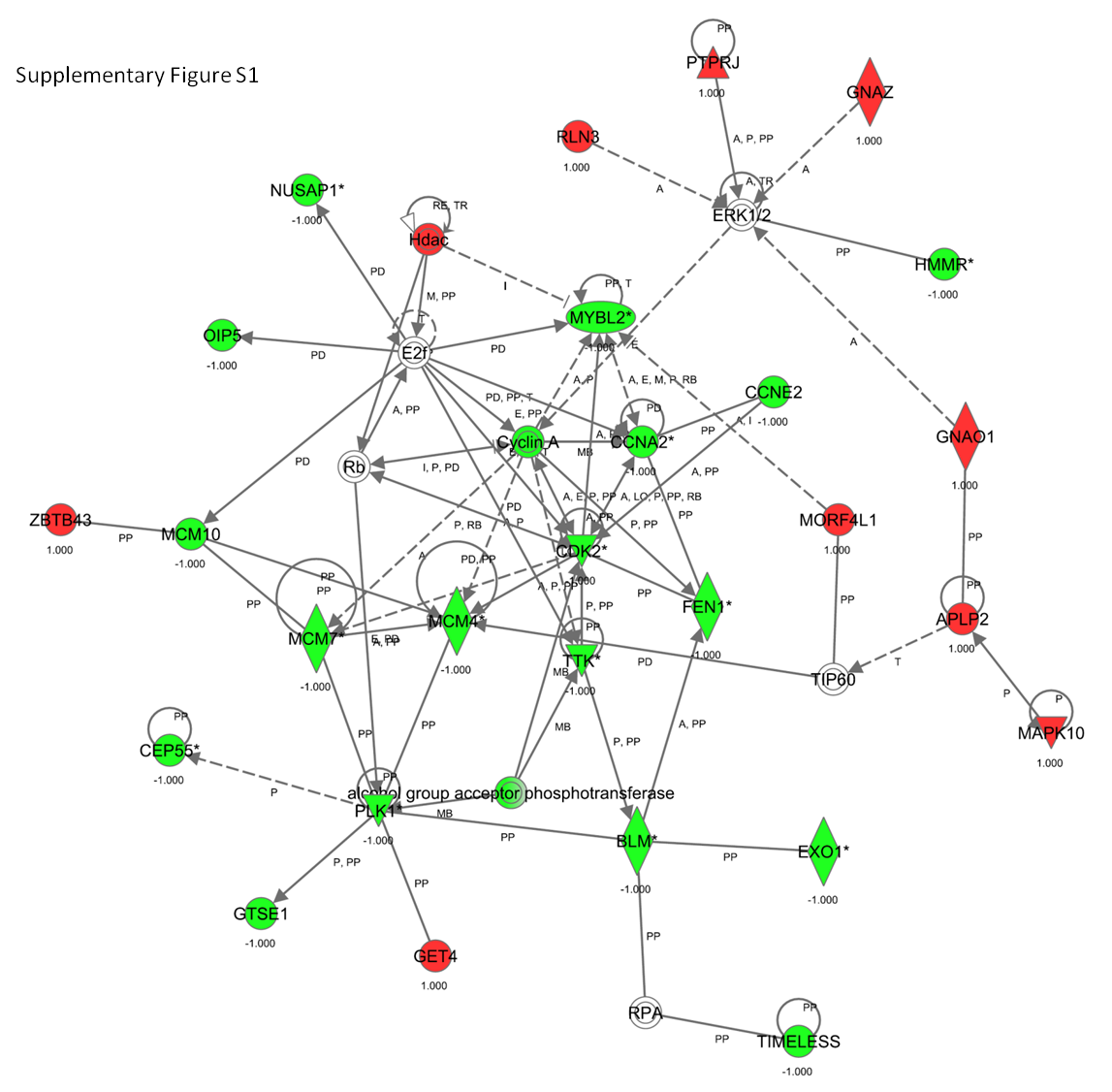

Supplement: Figure S1 — Gene network n°1 involving cell cycle genes from the analysis from IPA including the miR-210 target genes and GGI genes. (TIF) [file pone.0020980.s001.tif]

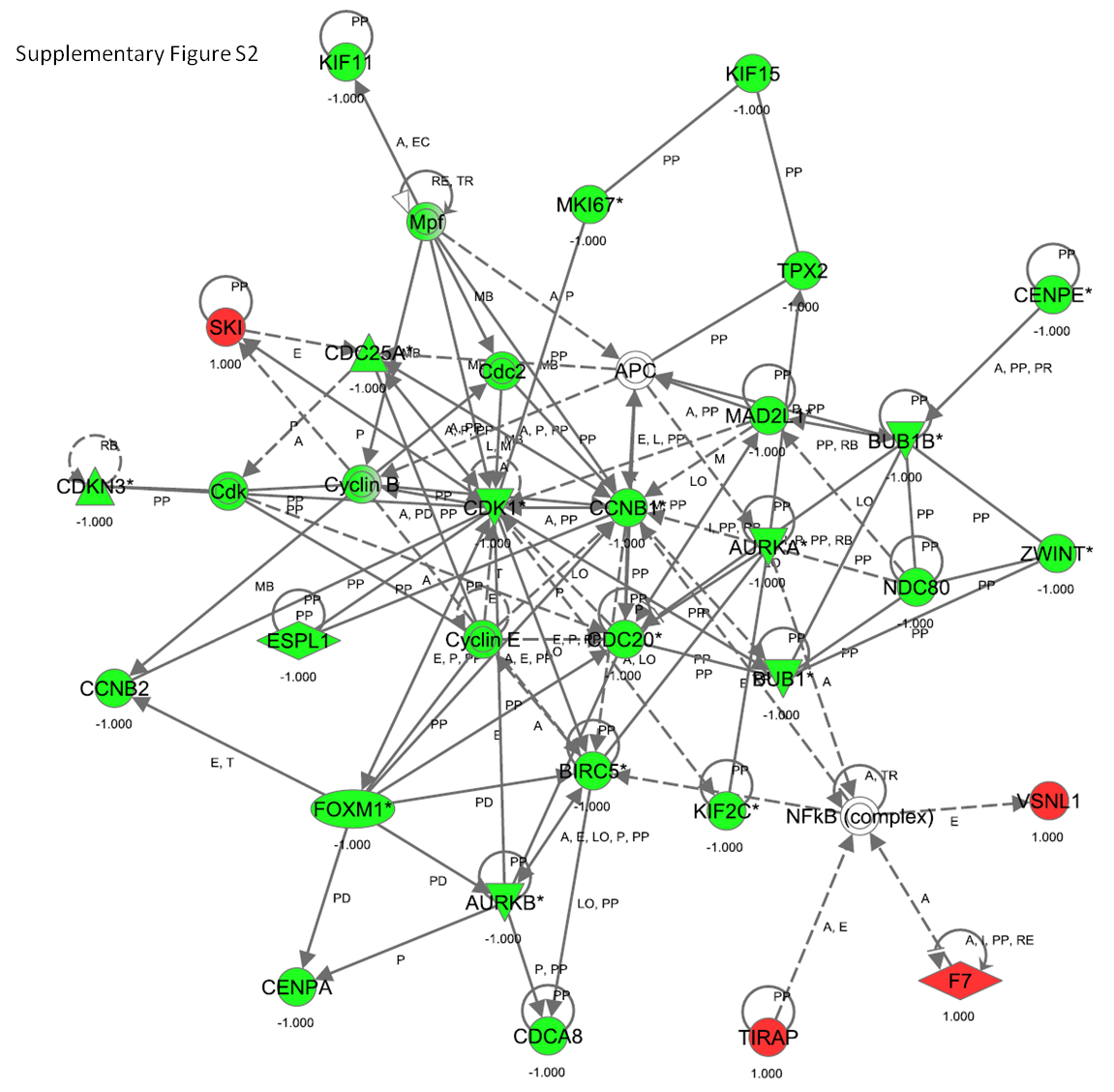

Supplement: Figure S2 — Gene network n°2 involving cell cycle genes from the analysis from IPA including the miR-210 target genes and GGI genes. (TIF) [file pone.0020980.s002.tif]

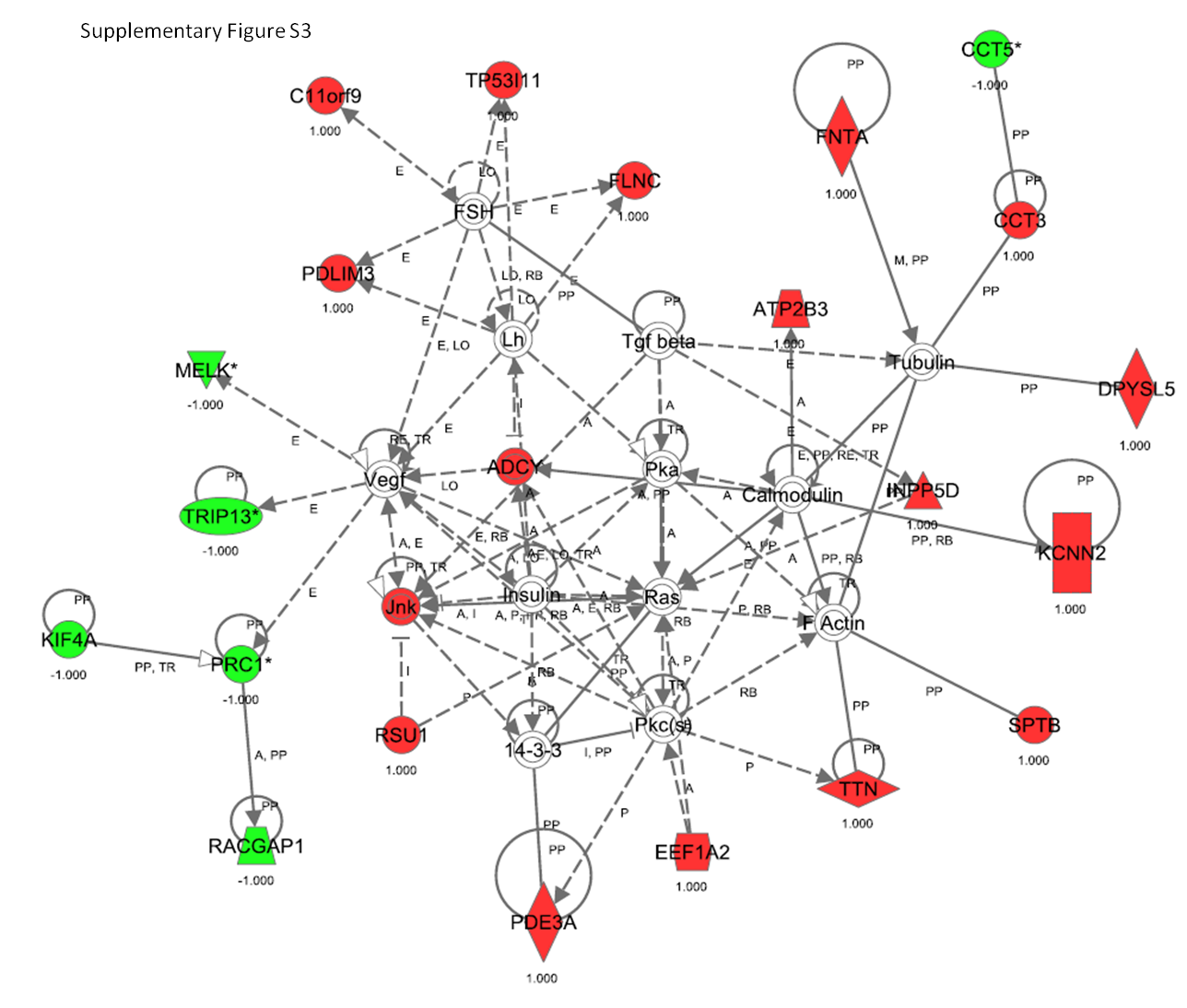

Supplement: Figure S3 — Gene network n°3 involving cell cycle genes from the analysis from IPA including the miR-210 target genes and GGI genes. (TIF) [file pone.0020980.s003.tif]

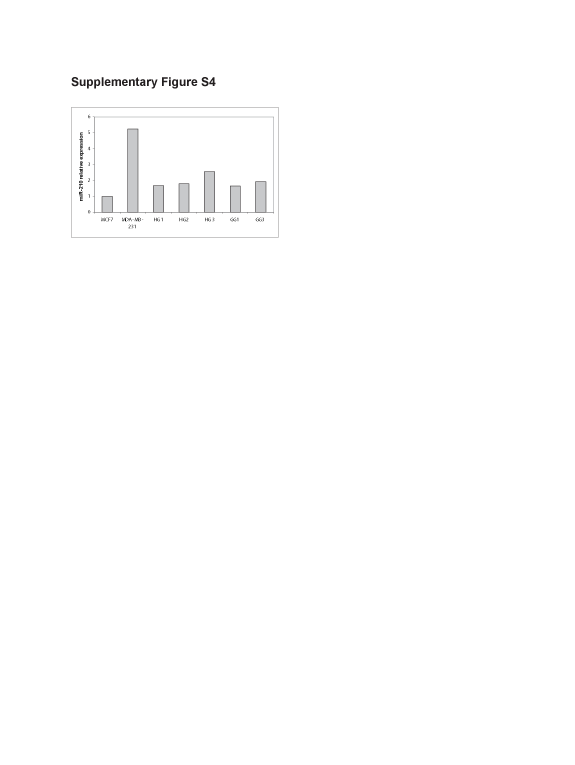

Supplement: Figure S4 — MiR-210 relative expression in MCF7 and MDA-MB-231 BC cell lines compared to BC samples according to histological grades (HG) and genomic grades (GG). (TIF) [file pone.0020980.s004.tif]

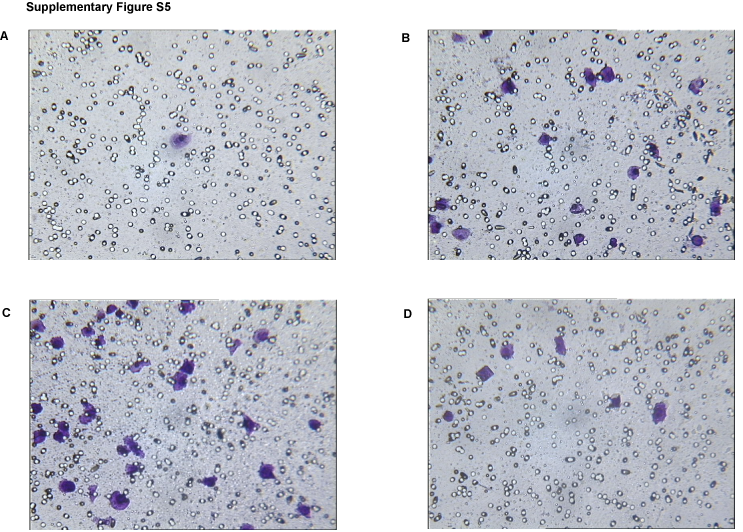

Supplement: Figure S5 — MiR-210 involvement in cell invasion and migration. MiR-210 overexpression enhances MCF7 cell invasion. Illustration of invading cells upon miR-210 overexpression compared to the control cells (A). MiR-210 repression enhances MDA-MB-231 cell migration. Illustration of migrating cells upon miR-210 repression compared to the control cells (B). (TIF) [file pone.0020980.s005.tif]
